# Evaluation of the diagnostic potential of antibodies to beta2-glycoprotein 1 domain 1 in Chinese patients with antiphospholipid syndrome

**DOI:** 10.1038/srep23839

**Published:** 2016-04-07

**Authors:** Shulan Zhang, Ziyan Wu, Si Chen, Jing Li, Xiaoting Wen, Liubing Li, Wen Zhang, Jiuliang Zhao, Fengchun Zhang, Yongzhe Li

**Affiliations:** 1 Department of Rheumatology and Clinical Immunology, Peking Union Medical College Hospital, Chinese Academy of Medical Sciences & Peking Union Medical College, Key Laboratory of Rheumatology and Clinical Immunology, Ministry of Education, No. 1 Shuai Fu Yuan, Eastern District, Beijing 100730, China; 2 Department of Clinical Laboratory, Beijing Anzhen Hospital, Capital Medical University, Beijing, 100029, China

## Abstract

In this study, we evaluated the clinical performance of anti-β2-glycoprotein 1 domain 1 antibodies (aβ2GP1-D1) in the diagnosis of antiphospholipid syndrome (APS). Sera from 229 subjects were tested, including 35 patients with primary APS, 51 patients with APS associated to other diseases, 30 patients with non-APS thrombosis, 32 patients with non-APS pregnancy-related morbidity, 42 patients with systemic lupus erythematosus, and 39 healthy controls (HC). Serum IgG aβ2GP1-D1, IgG/IgM anti-cardiolipin (aCL) and IgG/IgM aβ2GP1 were measured by a chemiluminescence assay. The levels of IgG aβ2GP1-D1 were significantly increased in patients with APS, compared with disease controls and HCs (*p* < 0.001). Significant correlation was identified between IgG aβ2GP1-D1 and IgG aβ2GP1 (p < 0.0001), indicating IgG aβ2GP1-D1 were the predominant domain-specific antibodies in IgG aβ2GP1 family. Importantly, aβ2GP1-D1, but not aβ2GP1 non-D1, was significantly correlated with thrombotic events. Interestingly, no significant correlation between IgG aβ2GP1-D1 and obstetric complications was observed. Additionally, significantly higher levels of IgG aβ2GP1-D1 were found in patients with triple aPL positivity, compared with patients with double and single aPL positivity. Our findings suggest a potential role of IgG aβ2GP1-D1 in identifying APS patients with high risk of thrombosis, shedding insight on the introduction of IgG aβ2GP1-D1 in China.

Antiphospholipid syndrome (APS) is an autoimmune disease characterized by recurrent thrombosis in arteries and veins and/or pregnancy morbidity. A hallmark feature of APS is the presence of the antiphospholipid antibodies (aPLs). aPLs represent a heterogeneous population of autoantibodies that target phospholipids, phospholipid-binding plasma proteins, and/or plasma protein-phospholipid complexes. Among those aPLs, anti-β2-glycoprotein 1 (aβ2GP1) antibodies have been increasingly recognized as the most clinically relevant autoantibodies in APS^1^. The presence of aβ2GP1, along with the presence of lupus anticoagulant (LA) and anticardiolipin antibodies (aCL), has been included in the standard diagnostic criteria for patients with clinical suspicion of APS^2^.

aβ2GP1 antibodies are a heterogeneous population. β2GP1 is composed of 5 domains. Domain 5 contains a phospholipid-binding site, which allows this domain to interact with the anionic phospholipids on the plasma membrane. This interaction leads to exposure of domain 1 (D1) into the extracellular space, making it possible to generate domain-specific antibodies for each of the 5 domains^3^. Indeed, the D1 epitope becomes available for antibody binding only when β2GP1 transitions from a circular form to a fish-hook conformation^4^.

*Iverson et al*. characterized the autoantibodies to each of the 5 β2GP1 domains and found that most aβ2GP1 antibodies reacted with epitope(s) in D1, indicating D1 as the main immunogenic epitope targeted by aβ2GP1 antibodies from patients with APS^5^,^6^,^7^. *De Laat et al*. further demonstrated that the Gly40-Arg43 region in β2GP1-D1 was the critical epitope, and antibodies against this region were able to interfere with the coagulation process and were strongly correlated with thrombosis^8^. Additionally, *Ioannou et al*. confirmed D1 as the immunodominant epitope of β2GP1 in animal models of aPL-induced thrombosis, as treatment of recombinant D1 peptide protected C57BL/6 mice from human aPL-induced pathology^9^. Subsequent work from several groups demonstrated that antibodies to β2GP1-D1 were associated with increased risk of thrombotic and obstetric manifestations in patients with APS^6^,^7^,^8^,^9^,^10^,^11^, suggesting that aβ2GP1-D1 antibodies could represent a pathogenic subpopulation of aβ2GPI antibodies.

Although aβ2GP1-D1 antibodies have attracted particular interest for its prognostic potential for thrombosis and pregnancy complications, the number of studies is still limited, and its clinical value needs to be verified in patients with different ethnic/geographic background. To our knowledge, few, if any, studies have reported the role of aβ2GP1-D1 antibodies in Chinese patients with APS. It is of paramount importance to evaluate this, as this information will enhance our understanding of the clinical utility of the aβ2GP1-D1 antibodies.

Currently, the detection of aβ2GP1-D1 antibodies is mainly based on ELISA assays with different detection strategies^6^. A novel chemiluminescence immunoassay (CIA) based assay for detecting aβ2GP1-D1 antibodies has recently developed. This novel CIA assay showed good agreements with ELISA^11^,^12^,^13^. We and others have showed that CIA assays have good performance in detecting aCL and aβ2GP1 autoantibodies^13^,^14^. More importantly, CIA has been considered as a promising tool to improve the reproducibility and reduce inter-laboratory variations. In this study, we utilized the CIA assay to evaluate the role of aβ2GP1-D1 antibodies in the diagnosis of APS, with a particular interest in their prognostic value for thrombosis and pregnancy complications.

## Results

### Levels of IgG aβ2GP1-D1 Antibodies were Elevated in Patients with APS

**Figure 1:**
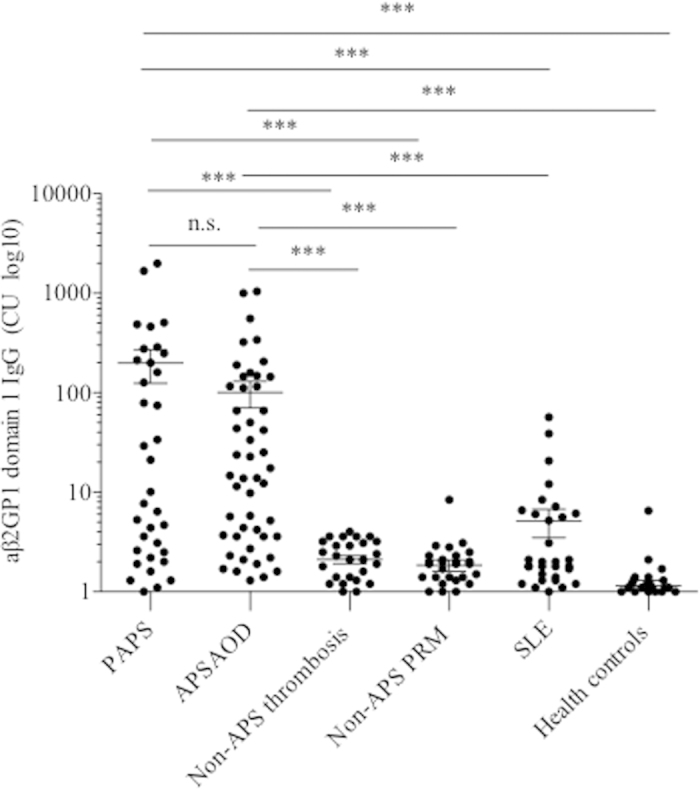
Levels of IgG aβ2GP1 D1 antibodies in Patients with APS and controls. The values expressed as CU of IgG aβ2GP1 D1 from PAPS, APSOD, Non-APS thrombosis, Non-APS RPM, SLE, and healthy controls. CU, chemiluminescent units; PAPS, primary APS; APSAOD, APS associated to other diseases; non-APS RPM, non-APS pregnancy-related morbidity, SLE, systemic lupus erythematosus. ***p < 0.001.

The values expressed as chemiluminescent units (CU) of IgG aβ2GP1-D1 from all subjects is presented in Fig. 1. The levels of IgG aβ2GP1-D1 were significantly increased in patients with APS, compared with patients with non-APS thrombosis, non-APS PRM, and SLE (p < 0.001), as well as healthy controls (p < 0.001). No significant difference in the levels of IgG aβ2GP1-D1 antibodies was observed between patients with PAPS and APSAOD. When the manufacturer’s recommended cut off of 20 CU was applied, the presence of IgG aβ2GP1-D1 antibodies in patients with PAPS, APSAOD, non-APS thrombosis, non-APS PRM, and SLE were 48.6%, 45.1%, 0, 0, and 7.1%, respectively (Table 1).

### Correlation between the Levels of IgG aβ2GP1-D1 Antibodies and the Levels of IgG aβ2GP1, IgG aCL Antibodies and LAC

**Figure 2:**
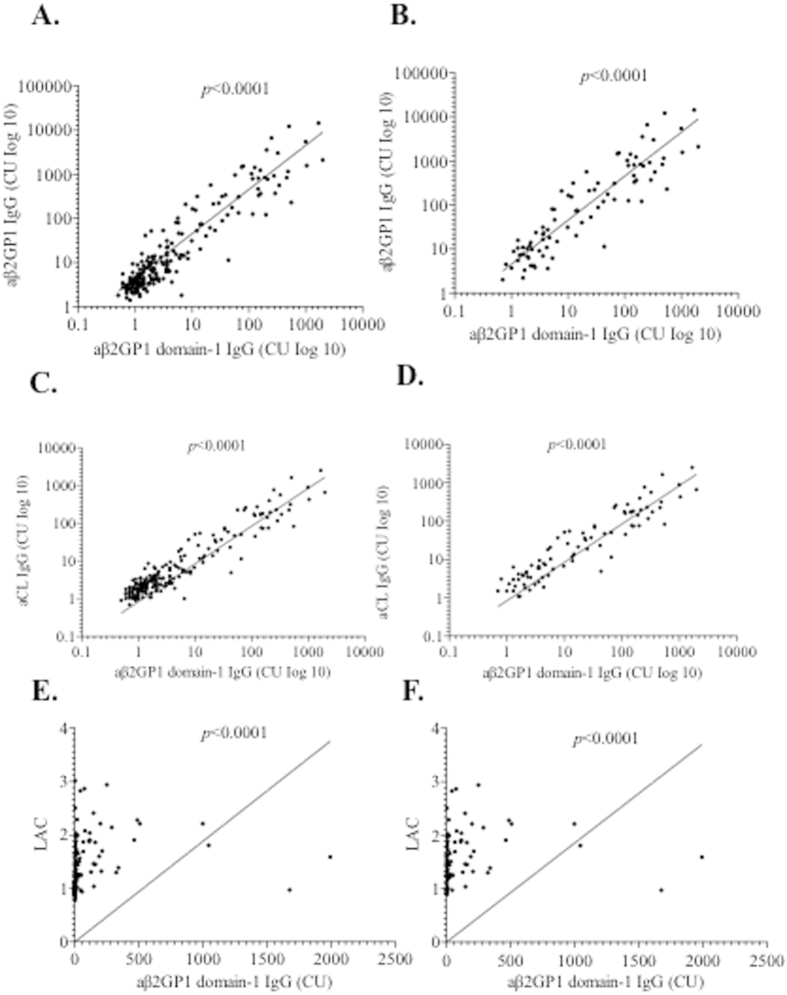
Correlation between levels of IgG aβ2GP1 D1 antibodies and the levels of IgG aβ2GP1 antibodies (**A,B**), between levels of IgG aβ2GP1 D1 antibodies and the levels of IgG aCL antibodies (**C,D**), and between levels of IgG aβ2GP1 D1 antibodies and the levels of LAC (**E,F**) in all subjects (**A,C,E**) and in patients with APS (**B,D,F**). The values expressed as CU of IgG aβ2GP1 and IgG aβ2GP1 D1. CU, chemiluminescent units; aCL, anticardiolipin antibodies; aβ2GP1, anti-β2-glycoprotein I antibodies; LAC, lupus anticoagulant.

As shown in Fig. 2, the levels of IgG aβ2GP1-D1 antibodies were significantly correlated with the levels of IgG aβ2GP1 antibodies in all subjects (p < 0.0001) (Fig. 2A) and in patients with APS (p < 0.0001) (Fig. 2B). Interestingly, 16 out of 86 APS patients (16/86, 18.6%) were positive for IgG aβ2GP1 and negative for IgG aβ2GP1-D1 (Fig. 2B). One out of 86 APS patients was positive for IgG aβ2GP1-D1 and negative for IgG aβ2GP1 (Fig. 2B). Notably, among the 16 APS patients that were positive for IgG aβ2GP1 and negative for IgG aβ2GP1-D1, the majority of the patients (13/16, 81.3%) only showed low levels of IgG aβ2GP1 (<100 CU) (Fig. 2B). In addition, significant correlations were also observed between the levels of IgG aβ2GP1-D1 antibodies and the levels of IgG aCL antibodies (Fig. 2C,D), and between levels of IgG aβ2GP1-D1 antibodies and LAC (Fig. 2E,F).

### Association between aβ2GP1-D1 Antibodies and Clinical Symptoms

It has been shown that aβ2GP1-D1 antibodies are associated with thromboembolic events, and to a lesser extent, with pregnancy morbidity^6^,^7^,^8^,^9^,^10^,^11^. Thus, the odds ratios (OR) were calculated to evaluate the association of aβ2GP1-D1 antibodies with those clinical manifestations in Chinese patients with APS. A significant association between aβ2GP1-D1 antibodies and thrombotic events was identified (OR, 3.27; 95% CI, 1.59–6.71) (Table 2). In addition, higher levels of aβ2GP1-D1 antibodies (>40 CU and >100 CU) were associated with higher OR values (4.48 and 3.67, respectively) (Table 2). In contrast, no significant associations were found between IgG aβ2GP1 non-D1 antibodies and thrombotic events (Table 2). Significant correlations were also observed between IgG aβ2GP1 and thrombotic events (OR, 2.75; 95% CI, 1.48–5.10), IgG aCL and thrombotic events (OR, 3.62; 95% CI, 1.77–7.41), and LAC and thrombotic events (OR, 3.28; 95% CI, 1.78–6.03) (Table 2). Interestingly, no significant correlation between IgG aβ2GP1-D1 antibodies and obstetric complications was observed (Table 2).

### Levels of IgG aβ2GP1-D1 antibodies in APS patients with different aPL profiles

**Figure 3:**
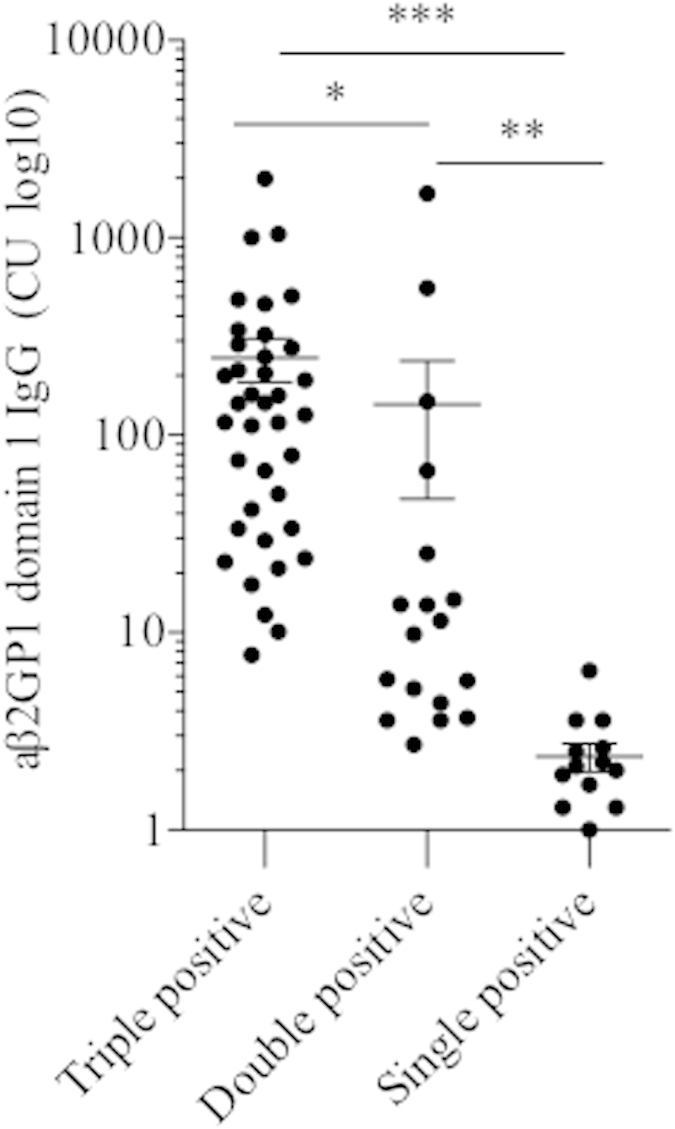
Levels of IgG aβ2GP1 D1 antibodies in triple (LAC+, IgG aCL+, IgG aβ2GP1+), double (LAC+/aCL+, LAC+/aβ2GP1+ or aCL+/aβ2GP1+), and single aPLs positive (positive for any of the aPLs) groups in patients with APS. The values expressed as CU of IgG aβ2GP1 D1 antibodies. CU, chemiluminescent units; aCL, anticardiolipin antibodies; aβ2GP1, anti-β2-glycoprotein I antibodies; LAC, lupus anticoagulant. *p < 0.05, **p < 0.01,***p < 0.001.

The levels of IgG aβ2GP1-D1 antibodies were also evaluated in APS patients with the triple aPL positivity, double aPL positivity, and single aPL positivity, as triple aPL positivity has been considered as a risk factor for aPL-mediated clinical manifestations^6^. Importantly, significantly higher levels of IgG aβ2GP1-D1 antibodies were found in patients with triple aPL positivity, compared with patients with double and single aPL positivity (Fig. 3). Additionally, patients with double aPL positivity exhibited significantly higher levels of IgG aβ2GP1-D1 antibodies, compared with patients with single aPL positivity (Fig. 3).

## Discussion

Previous studies have shown that abs specific to β2GP1-D1 are associated with thrombosis and pregnancy morbidity^6^,^7^,^8^,^9^,^10^,^11^. Two recent studies provide a proof of concept on the pathogenic role of aβ2GP1-D1 antibodies^15^,^16^. *Agostinis et al*. showed that a single-chain fragment variable (scFv) directed against β2GP1-D1 induced thrombosis and fetal loss in naïve rats/mice^15^. The other study demonstrated that aβ2GP1-D1-rich polyclonal IgG fractions from serum of patient with APS induced significantly larger thrombi *in vivo* compared with aDI-poor counterpart^16^. The pathogenic potential of aβ2GP1-D1 antibodies may come from their ability to trigger TLR4-NF-κB pathway, as β2GP1-D1 shares a high degree of homology with an extracellular epitope of human TLR4^17^. Given the significance of aβ2GP1-D1 antibodies, it is of paramount importance to characterize the clinical relevance of aβ2GP1-D1 antibodies in Chinese patients with APS.

In this study, we found that the levels of IgG aβ2GP1-D1 antibodies were significantly elevated in patients with APS. In addition, IgG aβ2GP1-D1 antibodies were the predominant domain-specific antibodies in IgG aβ2GP1 family. More importantly, aβ2GP1-D1 antibodies, but not aβ2GP1 non-D1 antibodies, were significantly correlated with thrombotic events. In contrast, no significant correlation between IgG aβ2GP1-D1 antibodies and obstetric complications was observed. Our findings suggest that aβ2GP1-D1 antibodies could serve as a promising biomarker to identify patients at risk of thrombosis in China.

We used the CIA assay in the entire study, rendering the results more reliable. Previously, we showed that the CIA assay had good performance characteristics and good agreements with a commercial ELISA from the same manufacturer^14^. As a variety of different assays have been used in detecting aβ2GP1-D1 antibodies (e.g., competitive inhibition ELISA with different D1 antigen, direct ELISA with different D1 antigen), the comparability of results across different studies might result in substantial variations^18^.

In the present study, IgG aβ2GP1-D1 antibodies were detected in 48.6% of patients with PAPS and 45.1% of patients with APSAOD. Mondejar *et al*. from Spain reported that IgG aβ2GP1-D1 antibodies were present in 31% of patients with PAPS and 46% of patients with APSAOD using the CIA assay^13^. The prevalence of IgG aβ2GP1-D1 antibodies in APSAOD patients was similar between the two studies, but the prevalence of IgG aβ2GP1-D1 antibodies in PAPS patients was higher in our study. Interestingly, a recent meta-analysis on 548 patients with APS from 11 different centers showed that the prevalence of IgG aβ2GP1-D1 antibodies was 44.0% (241/548)^19^, similar to what we found in this study.

It is worth mentioning that 13 patients with APS (7 patients with PAPS and 6 patients with APSAOD) were LAC positive but aβ2GP1 negative. This discrepancy may be due to the existence of other antibodies^20^. Indeed, when we tested those patients for anti-prothrombin/phosphatidylserine (aPS/PT) antibodies (QUANTA Lite^®^ aPS/PT, INOVA Diagnostic), 2 patients out of 7 (28.6%) with PAPS and 3 patients out of 6 (50.0%) with APSAOD exhibited positive for IgM aPS/PT antibodies (Zhang *et al*. unpublished data).

It has been suggested that the majority of the IgG aβ2GP1 antibodies bind to epitopes located in β2GP1-D^21^,^22^. In our study, we found IgG aβ2GP1-D1 antibodies were present in 81.4% of APS patients with positive aβ2GP1 antibodies, supporting D1 as the major epitope in β2GP1. Interestingly, in a multicenter study on patients with APS from Europe and the United States, IgG aβ2GP1-D1 antibodies were detected in 55% of patients with positive aβ2GP1 antibodies^10^, which is lower than that in our study. It is likely that the discrepancies are caused by different assays on IgG aβ2GP1 and IgG aβ2GP1-D1 antibodies detection, as we utilized the CIA assay, while they used the ELISA assay^10^. Interestingly, another study from Italy using CIA for IgG aβ2GP1 and IgG aβ2GP1-D1 determination showed that IgG aβ2GP1-D1 antibodies accounted for 69% of IgG aβ2GP1 antibodies^11^. Interestingly, one patient was negative for IgG aβ2GP1, but was positive for IgG aβ2GP1-D1. A possible explanation for this discrepancy could be due to the different epitopes recognized by IgG aβ2GP1 and IgG aβ2GP1-D1, as D1 epitope becomes available when β2GP1 transitions from a circular form to a fish-hook conformation^4^.

Multiple studies have highlighted a strong association between IgG aβ2GP1-D1 antibodies and thrombosis^6^,^7^,^8^,^10^,^11^,^13^. In this study, we found a significant correlation between IgG aβ2GP1-D1 antibodies with thrombotic events in Chinese patients with APS (OR, 3.27; 95% CI, 1.59–6.71). Strikingly, there was a further increase in the OR value when IgG aβ2GP1-D1 antibodies cutoff value was raised to >40 (OR, 4.48; 95% CI, 1.80–11.14). It is also noteworthy to mention that IgG aCL, IgG aβ2GP1, and LAC were also found significantly associated with thrombotic events in our study. *De Laat et al*. reported an OR of 3.5 (95% CI, 2.3–5.4) between IgG aβ2GP1-D1 antibodies and thrombotic events in an international multicenter study^10^, which is similar to what we observed. However, they only observed a week but significant association between LAC and thrombotic events (OR, 1.8; 95% CI, 1.1–3.1), and no association between aCL and thrombotic events (OR, 1.1; 95% CI, 0.6–2.1). As the patients from their study were selected based on positivity in the IgG/IgM aβ2GP1 ELISA, the bias might lead the results to favor the aβ2GP1 antibodies. Notably, antibodies to aβ2GP1-Domain 4/5 (D4/5) have also been characterized^23^,^24^,^25^,^26^. However, no associations were identified between aβ2GP1-Dm4/5 and thromboembolic events^27^. Interestingly, a recent study suggested that asymptomatic aPL carriers had higher levels of IgG aβ2GP1-D4/5, and an aβ2GP1-D1 to aβ2GP1- D4/5 ratio of ≥1.5 was predictive of systemic autoimmunity^28^. Thus, further studies are needed to determine the levels of IgG aβ2GP1-D4/5 in Chinese patients with APS using CIA, especially the ratio of aβ2GP1-D1 to aβ2GP1- D4/5.

Increasing evidence suggest that multiple positivity of aPLs are important parameters for risk assessment^29^,^30^. Interestingly, we observed that, 4 patients with PAPS had multiple thrombosis, and three of them exhibited triple-positive aPL profile and one showed positive LAC. Additionally, 3 patients with PAPS had both thrombosis and obstetric complications, and 2 of them exhibited triple-positive aPL profile and one showed positive LAC. In patients with APSAOD, 12 patients showed multiple thrombosis, and 5 of them showed triple-positive aPL profile, 5 of them displayed double-positive aPL profile, and the rest 2 patients exhibited positive LAC. In addition, 11 patients with APSAOD had both thrombosis and obstetric complications, and 5 of them exhibited triple-positive aPL profile, and the rest 6 patients showed double-positive aPL profile (data not shown). Notably, in patients with PAPS, 2 patients out of 35 (5.7%, one patient with LAC+/aCL+ and the other patient with aCL+/aβ2GP1+) exhibited double-positive aPL profile, while in patients with APSAOD, 15 patients out of 51 (29.4%, 11 patients with LAC+/aβ2GP1+ and 4 patients with aCL+/aβ2GP1+) showed double-positive aPL profile (data not shown). More importantly, in this study, we found significantly higher levels of IgG aβ2GP1-D1 antibodies in patients with triple-positive aPL profile, further supporting the importance of IgG aβ2GP1-D1 antibodies in evaluation of the APS clinical risks.

In contrast to the association between aβ2GP1-D1 antibodies with thrombosis, we did not observe any significant correlation between IgG aβ2GP1-D1 antibodies and obstetric complications, which differs from previous studies^6^,^7^,^8^,^10^,^11^. Moreover, no significant associations were observed between IgG aCL, IgG aβ2GP1, or LAC and obstetric complications. Different ethnic/geographic backgrounds might contribute to this discrepancy. Further studies with more APS patients with obstetric complications are needed.

It should be noted, however, that several limitations exist in this study. First, the diagnosis of patients with APS in this study requires the presence of at least one of the aPLs (LA, aCL, and aβ2GP1 autoantibodies)^2^, which might exclude the seronegative APS patients^31^. Second, we used sera from homogenous Chinese Han population. A multicenter study with different ethnic backgrounds is needed for generalizing our data to wider populations. Third, thrombosis is unusual in young non-APS subjects. Thus, patients with non-APS thrombosis were younger than patients with APS, as we wanted to reflect the real epidemiology in patients with non-APS thrombosis. Last, as mentioned before, more APS patients with obstetric complications are needed to assess the association of IgG aβ2GP1-D1 antibodies and obstetric complications.

In summary, our data suggest a potential role of IgG aβ2GP1-D1 antibodies in identifying APS patients with high risk of thrombosis, and thus could serve as a promising biomarker in clinical and therapeutic decision-making process. Our findings might shed insight on the introduction of IgG aβ2GP1-D1 antibodies in the laboratory diagnosis of APS in Chinese hospitals.

## Methods

### Subjects and Specimen Collections

Sera from 229 subjects were collected and analyzed in this study (Table 1). All the subjects were Chinese Han population. These subjects included 35 patients with primary APS (PAPS), 51 patients with APS associated to other diseases (APSAOD) (43 patients with SLE, 1 patient with both SLE and Sjögren’s syndrome (SS), 4 patients with connective tissue diseases, 1 patient with primary SS, 1 patient with Waldenstrom macroglobulinemia and 1 patient with tuberculous pleurisy), 30 patients with non-APS thrombosis, 32 patients with non-APS pregnancy-related morbidity (PRM), 42 patients with systemic lupus erythematosus (SLE), and 39 healthy controls (HC). HC were defined as no signs of infection or inflammation or other significant illnesses. APS was diagnosed according to the Sydney revised Sapporo guidelines^2^. Specifically, a combination of one positive clinical criterion and one positive laboratory criterion (LAC, aCL or aβ2G1 antibodies determined by ELISA) on two different occasions separated by 12 weeks were used for the diagnosis^2^. For patients with PAPS, treatments of patients at the time of serum collection include Aspirin (10/35, 28.6%), Warfarin (15/35, 42.9%), Heparin (7/35, 20.0%), Glucocorticoids (2/35, 5.7%), and Hydroxychloroquine (1/35, 2.9%). For patients with APSAOD, treatments of patients at the time of serum collection include Aspirin (19/51, 37.3%), Warfarin (21/51, 41.2%), Heparin (14/51, 27.5%), Glucocorticoids (24/35, 47.1%), and Hydroxychloroquine (15/51, 29.4%). The median time intervals between clinical events and the time of serum collection were 3 years (0.2–8 years) for patients with thrombosis and 1.5 years (0.3–6 years) for patients with obstetric complications. Clinical and laboratory features were collected from all the subjects. The presence of arterial and venous thrombosis in patients with PAPS, APSAOD, non-APS thrombosis, non-APS PRM, and SLE were 25.7% and 40.0%, 37.3% and 51.0%, 16.7% and 86.7%, 0 and 3.0%, and 2.3% and 0, respectively. The incidence of obstetric complications in patients with PAPS, APSAOD, non-APS thrombosis, non-APS PRM, and SLE were 52.6%, 51.4%, 0, 100%, and 0, respectively. LAC was determined by updated guidelines, as previously described^21^. The presence of LAC in patients with PAPS, APSAOD, non-APS thrombosis, non-APS PRM, and SLE were 71.4%, 78.4%, 6.7%, 3.1%, and 11.9%, respectively. Study protocols were reviewed and approved by the Ethical Committee of Peking Union Medical College Hospital (PUMCH) and informed consents were obtained from all participants. The study was conducted in accordance with the approved guidelines. All sera were stored at −20 °C until analysis.

### Serum aPL Antibodies Determination

Serum IgG and IgM aCL and IgG and IgM aβ2GP1 antibodies were determined by CIA (QUANTA Flash^®^ assays, INOVA Diagnostic, Inc, San Diego, CA) according to the manufacturer’s instructions, as previously described^14^,^32^. Serum IgG aβ2GP1-D1 antibodies were measured by CIA from QUANTA Flash^®^ β2GPI Domain 1 (INOVA Diagnostic, Inc, San Diego, CA). The principle and procedures of the QUANTA Flash^®^ β2GPI Domain 1 was previously described by *Pengo et al*.^14^. The cutoff values were set based on the recommendations by the manufacturer.

### Statistical Analysis

Prism 5.02 (GraphPad Software, San Diego, California, USA) was utilized for all statistical tests. Data of IgG aβ2GP1 (CU) and IgG aβ2GPI-D1 antibodies were transformed into log10 to create the Gaussian distribution. One-way ANOVA was used to calculate the difference between groups. Spearman’s correlation test was performed to analyze the correlation between IgG aβ2GP1 and IgG aβ2GPI-D1 antibodies. *p* values of less than 0.05 were considered statistical significant.

## Additional Information

**How to cite this article**: Zhang, S. *et al*. Evaluation of the diagnostic potential of antibodies to beta2-glycoprotein 1 domain 1 in Chinese patients with antiphospholipid syndrome. *Sci. Rep*. **6**, 23839; doi: 10.1038/srep23839 (2016).

## Figures and Tables

**Table 1:** Demographic, clinical characteristics and aCLs profiles of patients with APS and controls.

	PrimaryAPS (n = 35)	APS associated toother diseases (n = 51)	Non-APSthrombosis (n = 30)	Non-APSPRM (n = 32)	SLE controls(n = 42)	Health controls(n = 39)
Sex (female/male)	25/10	42/9	10/20	32/0	39/3	14/25
Median age at study (max, min)	34 (9, 76)	33 (5, 86)	53.5 (14, 85)	35 (24, 41)	30 (12, 68)	39 (25, 65)
Median duration/years (max, min)	1.5 (1, 6)	3 (1, 8)	3 (1, 11)	1 (1, 7)	4 (1, 21)	N/A
SLEDAI						
0–4 (%)	N/A	7 (15.9)****	N/A	N/A	6 (15.0)	N/A
5–9 (%)	N/A	7 (15.9)****	N/A	N/A	11 (26.0)	N/A
10–14 (%)	N/A	22 (50.0)****	N/A	N/A	12 (29.0)	N/A
≥15 (%)	N/A	8 (18.2)****	N/A	N/A	13 (31.0)	N/A
Arterial thrombosis, n (%)	9 (25.7)	19 (37.3)	5 (16.7)	0 (0.0)	1 (2.3)	0 (0.0)
Venous thrombosis, n (%)	14 (40.0)	26 (51.0)	26 (86.7)	1 (3.0)	0 (0.0)	0 (0.0)
Obstetric complications, n (%)*	10/19 (52.6)	18/35 (51.4)	0/10 (0.0)	32/32 (100.0)	0/31 (0.0)	0/14 (0.0)
aCL, n (%)**	22 (62.9)	28 (54.9)	0 (0.0)	1 (3.1)	3 (7.1)	0 (0.0)
aβ2GP1, n (%)**	19 (54.3)	38 (74.5)	0 (0.0)	2 (6.3)	8 (19.0)	1 (2.6)
LAC, n (%)	25 (71.4)	40 (78.4)	2 (6.7)	1 (3.1)	5 (11.9)	0 (0.0)
aβ2GP1 domain-1 IgG, n (%)	17 (48.6)	23 (45.1)	0 (0.0)	0 (0.0)	3 (7.1)	0 (0.0)
Non aβ2GP1 domain-1 IgG, n (%)***	2 (5.7)	12 (23.5)	0 (0.0)	1 (3.1)	5 (11.9)	0 (0.0)

*Percentage among married women of reproductive age. **IgG and/or IgM positive. ***aβ2GP1 IgG positive while aβ2GP1 domain-1 IgG negative. ****Percentage among patients with APSAOD with SLE. APS, antiphospholipid syndrome; RPM,pregnancy-related morbidity; SLE, systemic lupus erythematosus; SLEDAI, Systemic Lupus Erythematosus Disease Activity Index; aCL, anticardiolipin antibodies; aβ2GP1, anti-β2-glycoprotein I antibodies; LAC, lupus anticoagulant, N/A, not available.

**Table 2:** Correlations between aPLs and thrombosis or obstetrical complications in all patients.

aPLs	Odds ratio (95% confidence interval)	
	Thrombosis	Obstetrical complications
Anti-Domain Ι aβ2GP1 IgG (>20 CU)	3.27 (1.59–6.71)	1.55 (0.64–3.74)
Anti-Domain Ι aβ2GP1 IgG (>40 CU)	4.48 (1.80–11.14)	1.79 (0.60–5.38)
Anti-Domain Ι aβ2GP1 IgG (>100 CU)	3.67 (1.12–11.97)	1.52 (0.33–7.10)
Non-Domain Ι aβ2GP1 IgG	1.37 (0.57–3.28)	0.75 (0.27–2.12)
aβ2GP1 IgG	2.75 (1.48–5.10)	1.27 (0.60–2.67)
aβ2GP1 IgM	1.88 (0.51–6.87)	1.12 (0.22–5.79)
aCL IgG	3.62 (1.77–7.41)	1.96 (0.78–4.93)
aCL IgM	1.62 (0.58–4.55)	0.45 (0.11–1.83)
LAC	3.28 (1.78–6.03)	1.18 (0.57–2.46)
